# Peripheral blood eosinophils: an important reference for radiologists to distinguish between pulmonary paragonimiasis and tuberculous pleurisy in children

**DOI:** 10.1186/s12879-024-09461-3

**Published:** 2024-06-08

**Authors:** Kai-Yi Zhang, Yan Bi, Xu-Wen Fu, Min Qi, Jia-Lu Wei, Wei Gan, Le Zhang, Xiang Li

**Affiliations:** 1 Department of Tuberculosis, Kunming Third People’s Hospital/Yunnan Clinical Medical Center for Infectious Diseases, Kunming, 650041 China; 2 Department of Radiology, The People’s Hospital of Lincang, Lincang, Yunnan, 677000 China; 3 Department of Radiology, Kunming Third People’s Hospital/Yunnan Clinical Medical Center for Infectious Diseases, No. 319 of Wujing Street, Guandu District, Kunming, 650041 China; 4 Department of ICU, Kunming Third People’s Hospital/Yunnan Clinical Medical Center for Infectious Diseases, Kunming, 650041 China

**Keywords:** Diagnosis, Identification, Paragonimiasis, Pleura, Tomography, Tuberculosis, X-ray computer

## Abstract

**Objective:**

In this study, we examined the value of chest CT signs combined with peripheral blood eosinophil percentage in differentiating between pulmonary paragonimiasis and tuberculous pleurisy in children.

**Methods:**

Patients with pulmonary paragonimiasis and tuberculous pleurisy were retrospectively enrolled from January 2019 to April 2023 at the Kunming Third People’s Hospital and Lincang People’s Hospital. There were 69 patients with pulmonary paragonimiasis (paragonimiasis group) and 89 patients with tuberculous pleurisy (tuberculosis group). Clinical symptoms, chest CT imaging findings, and laboratory test results were analyzed. Using binary logistic regression, an imaging model of CT signs and a combined model of CT signs and eosinophils were developed to calculate and compare the differential diagnostic performance of the two models.

**Results:**

CT signs were used to establish the imaging model, and the receiver operating characteristic (ROC) curve was plotted. The area under the curve (AUC) was 0.856 (95% CI: 0.799–0.913), the sensitivity was 66.7%, and the specificity was 88.9%. The combined model was established using the CT signs and eosinophil percentage, and the ROC was plotted. The AUC curve was 0.950 (95% CI: 0.919–0.980), the sensitivity was 89.9%, and the specificity was 90.1%. The differential diagnostic efficiency of the combined model was higher than that of the imaging model, and the difference in AUC was statistically significant.

**Conclusion:**

The combined model has a higher differential diagnosis efficiency than the imaging model in the differentiation of pulmonary paragonimiasis and tuberculous pleurisy in children. The presence of a tunnel sign on chest CT, the absence of pulmonary nodules, and an elevated percentage of peripheral blood eosinophils are indicative of pulmonary paragonimiasis in children.

## Introduction

 Paragonimiasis is caused by the ingestion of metacercariae, which affects approximately 22.8 million people worldwide, primarily in Asia, Africa, and South America [1]. Tuberculosis is caused by infection with *Mycobacterium tuberculosis* in the human body. Clinically, pulmonary paragonimiasis is difficult to distinguish from active pulmonary tuberculosis due to the presence of cough, sputum production, fever, and pleural effusion [2]. On CT images, pulmonary paragonimiasis can manifest as pleural effusion as well as pulmonary nodules, strip-shaped, and with patchy consolidation, and is frequently misdiagnosed as tuberculosis and other diseases [2, 3]. When paragonimiasis is misdiagnosed as tuberculosis, patients are treated with anti-tuberculosis drugs, which not only prolong the condition of the patient but may also cause adverse drug reactions. Therefore, the differential diagnosis of the two diseases is of immense importance.

Laboratory tests represented by eosinophils have a certain value in the diagnosis of paragonimiasis and are able to differentiate between pulmonary paragonimiasis and tuberculous pleurisy [4]. In routine CT imaging, however, radiologists pay less attention to laboratory tests, and certain laboratory tests that can distinguish between the two are not popular. Therefore, in this study, we retrospectively collected the clinical features, imaging manifestations, and laboratory results of children with pulmonary paragonimiasis and tuberculous pleurisy, explored the differences between the two, and established a differential diagnostic model using CT imaging signs and highly accessible eosinophil levels and validated its efficacy, with the purpose of enhancing the understanding of the two diseases and improving the level of clinical differential diagnosis.

## Data and method

### Research participants

In this retrospective study, we examined children diagnosed with pulmonary paragonimiasis and tuberculous pleurisy between January 2019 and April 2023 at the Third People’s Hospital of Kunming and Lincang People’s Hospital.

Seventy-four children were diagnosed with paragonimiasis between January 2019 and April 2023 at the Third People’s Hospital of Kunming and Lincang People’s Hospital. Excluding 5 cases without pleural effusion (including 3 cases of cerebrospinal paragonimiasis and 2 cases of cutaneous paragonimiasis), a total of 69 patients with pulmonary paragonimiasis were enrolled in the paragonimiasis group. A total of 89 children with tuberculous pleurisy were selected; excluding 3 patients with co-bacterial infection and 5 patients not diagnosed for the first time, 81 children with tuberculous pleurisy were subsequently enrolled in the tuberculosis group.

There were 58 males and 11 females in the paragonimiasis group, with a median age of 9 (7–13) years, and there were 51 males and 30 females in the tuberculosis group, with a median age of 12 (9–13) years. The proportion of male patients in the paragonimiasis group (84.1%) was significantly higher than in the tuberculosis group (63.0%), and the median age was significantly lower than in the tuberculosis group (χ^2^ = 8.348, *P* = 0.004; *Z* = 2.534, *P* = 0.011).

### Inclusion and exclusion criteria

#### Inclusion criteria

Patients aged 0–14 years old, for the diagnostic criteria for paragonimiasis, we referred to *“WS 380–2012 Diagnosis of Paragonimiasis”* [5], and for the diagnosis of tuberculous pleurisy we referred to *“WS 288–2017 Diagnosis of Pulmonary Tuberculosis”* [6].

#### Exclusion criteria

Patients with other known infections, such as bacteria, fungi, viruses, and other pathogens; patients with paragonimiasis but without pleural effusion; patients who have previously received anti-tuberculosis or anti-parasitic therapy; patients who did not undergo a CT scan.

### Data collection

#### Clinical history and laboratory data

Collected through the hospital’s electronic record system.

#### Imaging examination

CT examination of patients were performed with uCT510 (Shanghai United Images), GE lightspeed VCT (U.S. GE), GE brightspeed (U.S. GE), and Aqulilon ONE TSX-301 C (Japan Canon), the scanning tube voltage was 120 kV, and the automatic milliampere technology was used for tube current, with a scanning layer thickness of 5 mm, and a reconstruction layer thickness of 0.625 to 1.25 mm; During the CT scan, a lead jacket was used to cover the patient’s non-target scanning site. Low-dose CT scan was preferred in all patients, unless enhanced CT was required. 

### Imaging analysis

The CT images were reviewed by two physicians with intermediate or higher level professional titles and with experience of several years in diagnosing infectious diseases using imaging, who were blinded. A third experienced physician reviewed and confirmed the diagnosis if there was any disagreement between the opinions of the two physicians, to reach a consensus.

For the diagnosis of certain CT signs, including consolidation, pleural effusion, cavitation, lymphadenopathy, and pulmonary nodules, the Fleischner Society’s glossary of chest radiology terminology was consulted [7]. The tunnel sign is characterized by the appearance of a tortuous, tunnel-like shadow with no distribution of lung texture in the patchy lung shadow. According to the chest CT findings, pleural effusions are classified into small, moderate, and large amounts.

### Statistical processing

SPSS 26.0 and Medcalc 20.0.1 software were used for statistical analysis. The measurement data were first tested for normality, and the data that conformed to normal distribution are described as mean ± standard deviation. An independent sample *t*-test was used for comparison. Data that conformed to skewed distribution are described as median “*M* (*Q*_*1*_, *Q*_*3*_)”, and were compared using the non-parametric test of two independent samples. The counting data are described using frequency and composition ratio/percentage (%), and the chi-squared test or Fisher’s exact probability test were adopted for comparison. The LR forward method was used in the binary logistic regression method to incorporate statistically significant variables from univariate analysis into the equation and the probability values of the equations were used for plotting the ROC. The DeLong test was used to compare the AUC of different ROC curves. *P* < 0.05 was considered statistically significant.

## Results

### Clinical symptoms

The incidence of chest pain and fever in the paragonimiasis group was significantly lower than that in the tuberculosis group, whereas the incidence of night sweats was significantly higher than that in the tuberculosis group (all *P* values < 0.05). The duration of symptoms in the paragonimiasis group was [30.0(15.0 ~ 90.0)] d, which was significantly longer than that in the tuberculosis group [20.0(10.0 ~ 60.0)] d (*P* = 0.025) (Table 1).

### Imaging manifestations

#### Pleural effusion

According to the site of occurrence, pleural effusion can be divided into bilateral and unilateral pleural effusion, and according to the amount of pleural effusion, it can be divided into small, medium, and large pleural effusion. Additionally, it can be classified as wrapped pleural effusion and free pleural effusion. The incidences of small, medium, and large pleural effusions and wrapped pleural effusions in the paragonimiasis group did not differ significantly from those in the tuberculosis group (*P* > 0.05); the incidence of bilateral pleural effusions in the paragonimiasis group was 34.8%, which was significantly higher than that in the tuberculosis group (11.1%) (all *P* values < 0.05) (Table 1).

#### Other manifestations

In addition to pleural effusion, pulmonary paragonimiasis and tuberculous pleurisy can be accompanied by pulmonary manifestations such as consolidation, pulmonary nodules, cavitation, and tunnel signs. There was no significant difference in the proportion of lung lobes affected between the two groups (all *P* values > 0.05); the incidence of tunnel signs was significantly higher in the paragonimiasis group than in the tuberculosis group, while the incidence of pulmonary nodules was significantly lower than that in the tuberculosis group(all *P* values < 0.05) (Table 1).

**Fig. 1:**
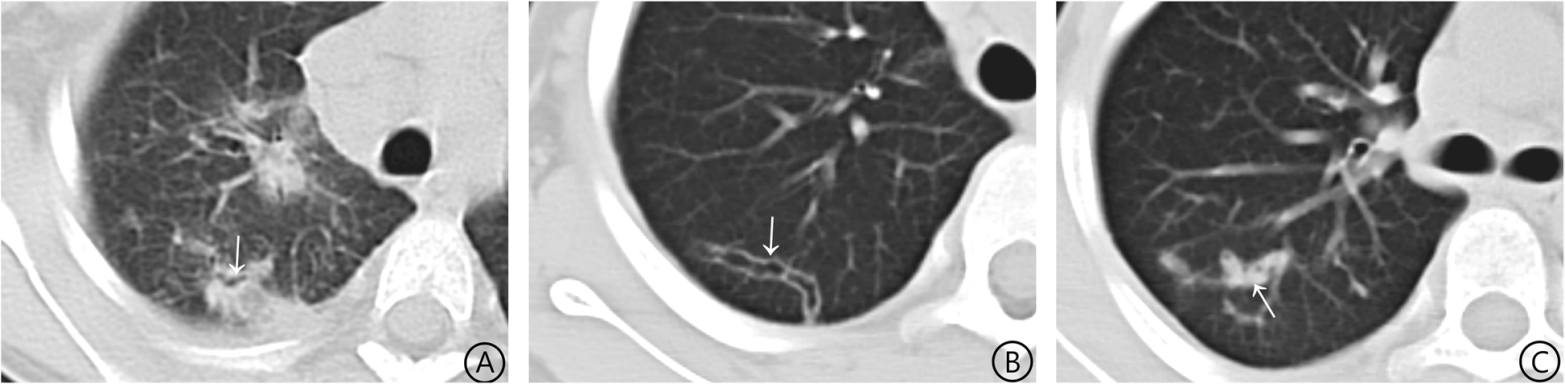
Figure 1 On CT, there are pulmonary tunnel signs in patients with paragonimiasis. **A** Multiple patchy consolidation shadows in both lungs, with tunnel signs (white arrow) within the consolidation shadows in the right upper lobe; **B** The consolidation surrounding the tunnel shadow of the right lung's upper lobe has been absorbed, leaving only a low-density tunnel shadow (white arrow); **C** Iso-density shadow completing the tunnel shadow of the right lung's upper lobe (white arrow)

The tunnel sign manifests as tunnel-like, low-density shadows in pulmonary consolidation and is most prevalent in the extrapulmonary belt (Fig. 1a). When the peripheral consolidation has been absorbed, it only appears as intrapulmonary low-density tunnel shadows (Fig. 1b), and some tunnels present with iso-density due to filling of cell debris or blood (Fig. 1c); it can also manifest as lymph node enlargement and pleural calcification.

**Fig. 2:**
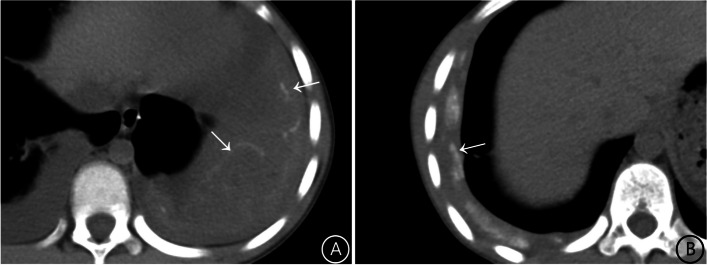
Pleural calcification on CT. **A** Paragonimiasis, bilateral pleural effusion, linear, map-like calcification of the left pleura (white arrow); **B** Tuberculous pleurisy, right pleural thickening, right pleural effusion, pleural manifestations of nodular and strip-like calcifications (white arrows)

Pleural calcification caused by paragonimiasis is typically characterized by patchy, irregular, and geographic patterns. (Figure 2a), whereas calcification caused by tuberculous pleurisy is predominantly strip-shaped or linear (Fig. 2b).

Incidence of pleural calcification was significantly higher in the paragonimiasis group (27.5%) compared to the tuberculosis group (8.6%). The incidence of lymph node enlargement was significantly lower in the paragonimiasis group (14.5%) than in the tuberculosis group (38.3%)(all *P* values < 0.05) (Table 1).

**Table 1:** A comparative analysis of the clinical symptoms and chest imaging findings of the two groups

Variables	Paragonimiasis group: (69 cases)		Tuberculosis group: (81 cases)		*P* value
	Cases	Incidence (%)	Cases	Incidence (%)	
Symptoms					
Cough	44	63.8	49	60.5	0.808
Sputum production	29	42.0	25	30.9	0.212
Chest pain	20	29.0	39	48.1	0.026
Chest tightness	17	24.6	32	39.5	0.078
Night sweats	19	27.5	9	11.1	0.018
Fever	29	42.0	52	64.2	0.011
Hemoptysis	6	8.7	1	1.2	0.049
Chest CT signs					
Consolidation	56	81.2	69	86.2	0.536
Cavitation	4	5.8	3	3.7	0.704
Pulmonary nodules	12	17.4	34	42.0	0.002
Tunnel sign	24	34.8	1	1.23	<0.001
Lung involvement areas					
Upper lobe of the right lung	23	33.3	26	32.1	1.000
Middle lobe of the right lung	24	34.8	25	30.9	0.737
Lower lobe of the right lung	30	43.5	44	54.3	0.246
Upper lobe of the left lung	24	34.8	22	27.2	0.406
Lower lobe of the left lung	39	56.5	36	44.4	0.190
Pleural effusion					
Small	35	50.7	40	49.4	0.967
Moderate	30	43.5	35	43.2	
Large	4	5.8	6	7.4	
Bilateral pleural effusion	24	34.8	9	11.1	0.001
Wrapped pleural effusion	32	46.4	47	58.0	0.208
Pleural calcification	19	27.5	7	8.6	0.005
Lymph node enlargement	10	14.5	31	38.3	0.002

### Laboratory examinations

#### Peripheral blood cell count

The total number of peripheral blood leukocytes, lymphocyte count, eosinophil count, and eosinophil percentage were significantly higher in the paragonimiasis group than in the tuberculosis group; however, the percentage of neutrophils in peripheral blood was significantly lower in the paragonimiasis group than in the tuberculosis group (all *P* values < 0.05) (Table 2).

**Table 2:** Comparative analysis of laboratory test results of the two groups

Indicators	Paragonimiasis group: (69 cases)	Tuberculosis group: (81 cases)	*P* value
Total number of leukocytes in peripheral blood (×10^9^/L)	12.4 [9.38;16.1]	6.43 [4.94;7.56]	< 0.001
Peripheral blood neutrophil count (×10^9^/L)	3.46 [2.81;4.41]	3.92 [2.60;4.98]	0.635
Peripheral blood lymphocyte count (×10^9^/L)	3.08 [2.35;4.29]	1.64 [1.30;2.07]	< 0.001
Peripheral blood eosinophil count (×10^9^/L)	3.95 [2.05;7.66]	0.09 [0.04;0.17]	< 0.001
Percentage of neutrophils (%)	33.4 [19.4;44.1]	58.7 [50.3;66.6]	< 0.001
Percentage of lymphocytes (%)	27.0 ± 9.88)	28.6 ± 10.8	0.324
Percentage of eosinophils (%)	33.5 [20.5;49.0]	1.38 [0.61;2.85]	< 0.001
Erythrocyte sedimentation rate (mm/1 h)	35.0 [15.0;43.0]	31.0 [16.0;40.0]	0.613
Total serum protein (g/L)	74.5 [69.0;78.5]	66.9 [62.3;71.2]	< 0.001
Serum albumin (g/L)	35.4 [32.7;38.5]	35.2 [30.9;38.1]	0.581
Serum globulin (g/L)	37.4 [32.8;44.2]	32.1 [27.9;35.3]	< 0.001
Serum albumin/globulin ratio	0.95 ± 0.26	1.11 ± 0.29	< 0.001

#### Plasma protein detection

The serum total protein and globulin levels were significantly higher in the paragonimiasis group than in the tuberculosis group, whereas the serum albumin/globulin ratio was significantly lower in the paragonimiasis group than in the tuberculosis group(all *P* values < 0.05) (Table 2).

### Differential diagnosis model establishment and model efficiency

#### Establishment of imaging and combined models

On CT images, the variables pulmonary nodules, tunnel signs, pleural calcifications, lymph node enlargement, and bilateral pleural effusion were used to establish a differential diagnosis model using the forward LR method and binary logistic regression. The regression equation was logit(P) = 1.751 + 2.361×pulmonary nodules-5.179×tunnel sign-1.452×pleural calcification-1.34×bilateral pleural effusion. With patients without pulmonary nodules as the control, pulmonary nodules were more likely to appear in patients with tuberculous pleurisy (*OR* = 10.603, 95% *CI*: 3.045–53.87). With patients without tunnel signs as the control, tunnel signs were less likely to appear in patients with tuberculous pleurisy (*OR* = 0.006, 95% *CI*: 0.00-0.045). With patients without pleural calcification as the control, pleural calcification was less likely to appear in patients with tuberculous pleurisy (*OR* = 0.234, 95% *CI*: 0.07–0.706). With patients without lymph node enlargement as the control, lymph node enlargement was more likely to appear in patients with tuberculous pleurisy (*OR* = 2.793, 95% *CI*: 1.024–8.361). With patients with unilateral pleural effusion as the control, bilateral pleural effusion was less likely to appear in patients with tuberculous pleurisy (*OR* = 0.262, 95% *CI*: 0.086–0.734) (Table 3).

**Table 3:** Multivariate regression analysis of imaging models

Imaging signs	B	SE	OR	CI	Z	*P*
Constant	1.751	0.69407	5.758	5.758(1.51–23.31)	2.522	0.012
Pulmonary nodules	2.361	0.71406	10.603	10.60(3.045–53.87)	3.307	0.001
Tunnel sign	-5.179	1.25926	0.006	0.006(0-0.045)	-4.113	0
Bilateral pleural effusion	-1.34	0.54119	0.262	0.262(0.086–0.734)	-2.476	0.013
Pleural calcification	-1.452	0.58222	0.234	0.234(0.07–0.706)	-2.494	0.013
Lymph node enlargement	1.027	0.52949	2.793	2.793(1.024–8.361)	1.94	0.052

Based on the normal value (0.5–5%), the percentage of eosinophils was divided into elevated and non-elevated groups to facilitate clinical application. Using pulmonary nodules, tunnel signs, pleural calcifications, lymph node enlargement, and bilateral pleural effusion on CT images, as well as an elevated percentage of eosinophils, the forward LR method was used to develop a differential diagnosis model. The regression equation was logit(P) = 2.251 + 2.357×pulmonary nodules-4.164×tunnel signs-4.01×elevated eosinophil percentage. With patients without pulmonary nodules as the control, pulmonary nodules were more likely to appear in patients with tuberculous pleurisy (*OR* = 10.555, 95% *CI*: 2.509–58.08). With patients without tunnel signs as the control, tunnel signs were less likely to appear in patients with tuberculous pleurisy (*OR* = 0.016, 95% *CI*: 0.001–0.155). With patients with normal eosinophil percentage as the control, elevated eosinophil percentage was less likely to appear in patients with tuberculous pleurisy (*OR* = 0.018, 95% *CI*: 0.005–0.055) (Table 4).

**Table 4:** Multivariate regression analysis of the combined model

Combined model parameters	B	SE	OR	CI	Z	*P*
Constant	2.215	0.47784	9.162	9.162 (3.953–26.75)	4.636	0
Elevated percentage of eosinophils	-4.01	0.61031	0.018	0.018 (0.005–0.055)	-6.571	0
Pulmonary nodules	2.357	0.78469	10.555	10.55 (2.509–58.08)	3.003	0.003
Tunnel sign	-4.164	1.40054	0.016	0.016 (0.001–0.155)	-2.973	0.003

#### Efficacy and comparison of imaging model and combined model

**Fig. 3:**
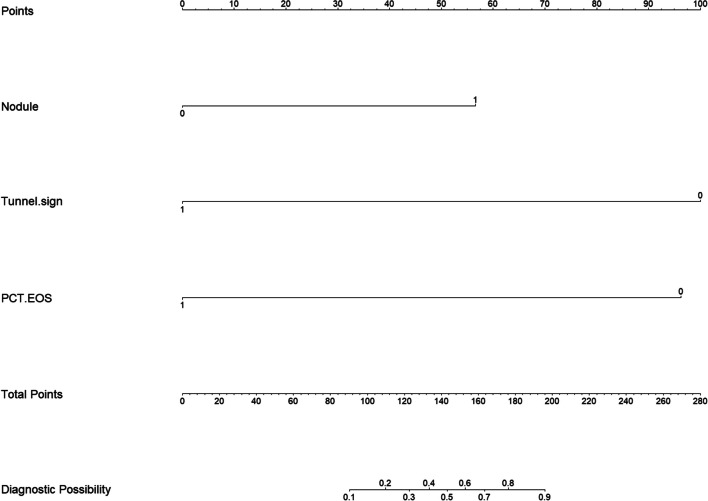
Figure 3 NOMO plot of combined model. Nodule: Pulmonary nodules (0 = no, 1 = yes). Tunnel sign: Tunnel sign (0 = no, 1 = yes). PCT.EOS: Percentage of eosinophils (0 = no, 1 = yes)

Efficacy and comparison of imaging model and combined model: ROC was plotted using imaging model and combined model. The imaging model AUC was 0.856 (95% *CI*: 0.799, 0.913), with a specificity of 88.9% and a sensitivity of 66.7%. The combined model AUC was 0.950 (95% *CI*: 0.919, 0.980), with a specificity of 90.1% and a sensitivity of 89.9%; The DeLong test was used to compare the efficacy of the two models in the differential diagnosis of paragonimiasis and tuberculous pleurisy in children. The AUC of the combined model was significantly higher than that of the imaging model (Table 5), and the NOMO plot of combined model was established (Fig. 3).

**Table 5:** Differential diagnostic performance of imaging and combined models

Model	AUC (95%CI)	Sensitivity (%)	Specificity (%)
Imaging model	0.856 (0.799～0.913)	66.7	88.9
Combined model	0.950 (0.919～0.980)	89.9	90.1

## Discussion

Paragonimus is a species of lung fluke that infects humans when they consume its metacercaria, causing paragonimiasis. In China, lung flukes are common in Yunnan, Sichuan, Chongqing, and other places [8]. Multiple organs such as the lungs, brain, spinal cord, skin, abdominal cavity can be infected by a paragonimus infection, and the infections can be divided into the pulmonary type, cerebrospinal cord type, skin type, abdominal type and mixed type [9]. Tuberculosis is caused by *M. tuberculosis* infection in humans, and is a major public health problem. There is a high prevalence of tuberculosis in Yunnan Province in China, and its incidence is expected to increase [10]. Currently, epidemiological data on paragonimiasis and tuberculous pleurisy in children in Yunnan Province have rarely been reported.

During clinical diagnosis and treatment, pulmonary paragonimiasis and tuberculous pleurisy in children may manifest as fever, night sweats, cough, sputum production, chest pain, chest tightness, and additional systemic or respiratory symptoms. Although in our study, the incidences of fever and chest pain in children with tuberculosis were higher than those in children with pulmonary paragonimiasis, the differential diagnosis during the specific first diagnosis process in clinical practice remains challenging. In this study, the proportion of males with pulmonary paragonimiasis was significantly higher than that of children with tuberculosis, while the median age of children with tuberculosis was slightly lower. This disparity may be attributable to the transmission route of paragonimus. Earlier research revealed that the consumption of raw or semi-raw stream crabs containing the fluke metacercaria has been linked to the occurrence of paragonimiasis in elementary school boys in Yunnan Province [11]. 

With the advancement of medical imaging technology and equipment, the radiation dose of CT equipment has gradually decreased, and CT scans are now commonly used to examine children for chest diseases. However, considering that CT is the largest medical source of radiation exposure [12], and results from large-scale epidemiologic studies predicted an increased risk of cancer associated with exposure to CT radiation among radiosensitive children and adolescents [13]. Low-dose CT protocol has been confirmed by a large number of studies, which can significantly reduce ionizing radiation on the premise of ensuring image quality [12, 14]. For most cases in our study, low-dose CT protocol was used to evaluated the lesions to perform radioprotection in children. Chest CT findings of pulmonary paragonimiasis may include pleural effusion, pleural hypertrophy calcification, pulmonary nodules, cystic changes, and tunnel signs [15]. Tunnel signs are the lung tissue damage caused by the migration of paragonimus in the lungs; it is a typical imaging manifestation of pulmonary paragonimiasis [16]. Due to the oligo-bacterial nature of pediatric tuberculosis, imaging plays a key role in the diagnosis, and tuberculous pleurisy can manifest as pleural effusion, wrapped pleural effusion, pleural adhesions, pleural calcification, and so on [17]. More than 30% of tuberculous pleural effusions are wrapped, which may be related to the conversion of fibrinogen to fibrin [18]. Approximately 50% of children with tuberculosis have two or more concurrent tuberculosis imaging changes, including consolidation, cavitation, pulmonary nodules, and bronchial stenosis in addition to pleural lesions [19]. The location of pulmonary lesions, intrapulmonary consolidation, and cavitation were not statistically significant between the two diseases in this study. Although the tunnel sign is a characteristic imaging manifestation of pulmonary paragonimiasis, some worm-eaten cavities caused by tuberculosis could be misinterpreted as tunnel signs, and the percentage of tunnel signs in pulmonary paragonimiasis is only 34.8%. Although the incidences of bilateral pleural effusion, pleural calcification, and lymph node enlargement were significantly different between the two groups, none of these were diagnostically different. In this study, an imaging model was developed using the above statistically significant indicators and binary logistic regression, and its differential diagnostic efficacy was tested. The AUC was 0.856, and the sensitivity and specificity were 66.7% and 88.9%, respectively. False negatives and false positives remain in the differential diagnosis of CT signs.

*Paragonimus westermani* are the leading cause of elevated peripheral blood eosinophils among human parasitic infections [20]. However, some children diagnosed with tuberculosis can still exhibit elevated eosinophils [4], which complicates the clinical diagnosis. In this study, the total number of peripheral blood leukocytes, eosinophil count, and eosinophil ratio of children with pulmonary paragonimiasis were significantly higher than those of children with tuberculous pleurisy, as was the globulin level. Thus, the CT imaging signs and laboratory tests were combined to develop a differential diagnostic model. To facilitate clinical use, it was divided into dichotomous variables based on whether the proportion of eosinophils was elevated. The binary logistic regression model was used to establish the combined model, with the AUC value of 0.950 and sensitivity and specificity values of 89.0% and 90.1%, respectively. The efficacy of the imaging model and the combined model in the differential diagnosis of paragonimiasis in children and tuberculous pleurisy in children was compared using the DeLong test, and the AUC value of the combined model was significantly higher than that of the imaging model. After establishing the differential diagnosis model, the corresponding differential nomogram was plotted based on the model, which is convenient for clinical use and can improve the diagnostic compliance rate.

## Conclusion

In conclusion, in the process of clinical diagnosis and treatment, special attention should be paid to the observation of bilateral pleural effusion, pleural calcification, pulmonary nodules, lymph node enlargement, and other chest CT signs for differential diagnosis of the aforementioned two diseases. In addition, it is critical to carefully observe for the presence of a tunnel sign resulting from the migration of paragonimus in the lungs. If there are difficulties in imaging differentiation, radiologists can examine the proportion of peripheral blood eosinophils in the patient and use the equation of the combined model to differentiate between the two.

## Data Availability

The datasets used and/or analysed during the current study available from the corresponding author on reasonable request.

## References

[1] Shah P, Sah R, Pradhan S (2023). Pulmonary paragonimiasis: A case series. JNMA J Nepal Med Assoc.

[2] Morter R, Adetifa I, Antonio M (2018). Examining human paragonimiasis as a differential diagnosis to tuberculosis in the Gambia. BMC Res Notes.

[3] Villanueva-Villegas R, Diaz-Mendoza J, Salas-Lopez J (2023). Paragonimiasis misdiagnosed as pulmonary tuberculosis: A Case Report. Cureus.

[4] Poudyal BS, Paudel B, Bista B (2022). Clinical, Laboratory and Radiological features of Paragonimiasis misdiagnosed as pulmonary tuberculosis. Iran J Parasitol.

[5] General Administration of Quality Supervision (2012). Inspection and Quarantine of the China, Standardization Administration of China.

[6] General Administration of Quality Supervision, Inspection and Quarantine of the China, Standardization Administration of China. WS 288-2017 Diagnostic criteria for pulmonary tuberculosis. Standards Press of China. 2017-11-09.

[7] Hansell DM, Bankier AA, MacMahon H (2008). Fleischner Society: glossary of terms for thoracic imaging. Radiology.

[8] Jiang YX, Li GQ, Pan CJ (2023). Pediatric paragonimiasis: a retrospective analysis of cases from a county in south-west China. Front Pediatr.

[9] Yoshida A, Doanh PN, Maruyama H (2019). Paragonimus and paragonimiasis in Asia: an update. Acta Trop.

[10] Chen J, Ruan Y, Wang K (2022). Development and application of a Multidrug-Resistant Tuberculosis Case Management System - Yunnan Province, China, 2017–2020. China CDC Wkly.

[11] Li X, Fu X, Geng P (2021). Clinical and imaging analysis for paragonimiasis in children in Yunnan Province. J Trop Dis Parasitol.

[12] Nagayama Y, Oda S, Nakaura T (2019). Radiation dose reduction at pediatric CT: use of low tube voltage and iterative Reconstruction. Radiographics.

[13] Miglioretti DL, Johnson E, Williams A (2013). The use of computed tomography in pediatrics and the associated radiation exposure and estimated cancer risk. JAMA Pediatr.

[14] Nekolla EA, Brix G, Griebel J (2022). Lung cancer screening with low-dose CT: radiation risk and benefit-risk assessment for different screening scenarios. Diagnostics (Basel).

[15] Li KK, Jin GY, Kwon KS (2022). What findings on chest CTs. Can Delay Diagnosis Pleuropulmonary Paragonimiasis?. Tomography.

[16] Tonne EO, Fosbøl MØ, Poulsen A (2022). Imaging modalities for pulmonary tuberculosis in children: a systematic review. Eur J Radiol Open.

[17] Lee J, Park J, Park JE (2023). Different characteristics of pleural abnormalities on computed tomography between tuberculous and malignant pleural effusions. Am J Med Sci.

[18] Yamamoto J, Nishiura M, Ohata T (2018). Tuberculous pleurisy diagnosed by Thoracoscopic Lung Biopsy. Kyobu Geka.

[19] Naranje P, Bhalla AS, Sherwani P (2019). Chest tuberculosis in children. Indian J Pediatr.

[20] Sakakura S, Yamaguchi F, Abe T (2023). Pneumothorax with Eosinophilia is an important Diagnostic Clue for distinguishing paragonimiasis from chronic eosinophilic pneumonia: A Case Report. Infect Drug Resist.

